# Severe falciparum malaria in Gabonese children: clinical and laboratory features

**DOI:** 10.1186/1475-2875-4-1

**Published:** 2005-01-09

**Authors:** Arnaud Dzeing-Ella, Pascal C Nze Obiang, Rose Tchoua, Timothy Planche, Béatrice Mboza, Monique Mbounja, Ulrich Muller-Roemer, Joseph Jarvis, Eric Kendjo, Edouard Ngou-Milama, Peter G Kremsner, Sanjeev Krishna, Maryvonne Kombila

**Affiliations:** 1 Department of Parasitology, Mycology and Tropical Medicine, Faculty of Medicine, University of Health Sciences (USS), Libreville, Gabon; 2 Department of Paediatrics, Centre Hospitalier de Libreville (CHL), Gabon; 3 Malaria Clinical Research Unit, CHL, Gabon; 4 Department of Infectious Diseases, St. George's Hospital Medical School, Cranmer Terrace, London, UK; 5 Department of Intensive Care-Emergency, CHL, Gabon; 6 Department of Parasitology, Eberhard Karls Universität, Tübingen, Germany; 7 Department of Biochemistry, Faculty of Medicine, USS, Libreville, Gabon

## Abstract

**Background:**

Malaria continues to claim one to two million lives a year, mainly those of children in sub-Saharan Africa. Reduction in mortality depends, in part, on improving the quality of hospital care, the training of healthcare workers and improvements in public health. This study examined the prognostic indicators of severe falciparum malaria in Gabonese children.

**Methods:**

An observational study examining the clinical presentations and laboratory features of severe malaria was conducted at the Centre Hospitalier de Libreville, Gabon over two years. Febrile children aged from 0 to 10 years with *Plasmodium falciparum *infection and one or more features of severe malaria were enrolled.

**Results:**

Most children presenting with severe falciparum malaria were less than 5 years (92.3% of 583 cases). Anaemia was the most frequent feature of severe malaria (67.8% of cases), followed by respiratory distress (31%), cerebral malaria (24%) hyperlactataemia (16%) and then hypoglycaemia (10%). Anaemia was more common in children under 18 months old, while cerebral malaria usually occurred in those over 18 months. The overall case fatality rate was 9%. The prognostic indicators with the highest case fatality rates were coma/seizures, hyperlactataemia and hypoglycaemia, and the highest case fatality rate was in children with all three of these features.

**Conclusions:**

Prompt and appropriate, classification and treatment of malaria helps identify the most severely ill children and aids early and appropriate management of the severely ill child.

## Introduction

Each year 500 million infections and up to 2.7 millions deaths are attributable to malaria [1], about 90% of these deaths occur in children in sub-Saharan Africa [2]. Eighty percent of the deaths occur during the first 24 hours following admission [3-5]. Despite a better understanding of pathophysiology and management of malaria, childhood mortality remains unacceptably high [6]. The acquisition of malaria immunity is closely linked to the level of transmission and severe *Plasmodium falciparum *infection is very rare after the age of 5 years in highly endemic areas. Presentations of severe malaria are different at different ages and in areas with different levels of transmission. In Gabon, a country of about 1.2 million people, malaria is the main cause of neurological, haematological and infectious emergencies at the Centre Hospitalier de Libreville (CHL), the country's tertiary referral centre [7]. Malaria transmission is hyperendemic and perennial with an entomological inoculation rate of 50 per person-year [8]. Previous studies in Africa have shown three frequent presentations of severe falciparum malaria: cerebral malaria, metabolic malaria (hyperlactaemia, acidosis or respiratory distress) and severe anaemia [3,5,9]. The case fatality rate of severe anaemia, however, is low and in some studies it is not an independent predictor of death [9,10]. In Africa, malaria mortality remains high for a number of reasons including limited access to healthcare and increased drug resistance [6]. Better classification of severe malaria could aid clinicians caring for children with severe malaria to avoid diagnostic delays, identify the children most likely to die and thus improve management by targeting resources to the sickest children. This prospective observational study was designed to determine the clinical and laboratory features that identify those children most severely ill with malaria.

## Methods

This study was carried out in the CHL between 1st August 2000 and 31st July 2002. The CHL is the largest public hospital in the country, situated in Libreville, the capital city (pop. 500,000). Services at the CHL include an Emergency Department (20 beds), a Paediatrics Unit (80 beds) and an Intensive Care Unit (12 beds). Ethical permission for the study was granted by the Gabonese Ministry of Health.

**Figure 1:**
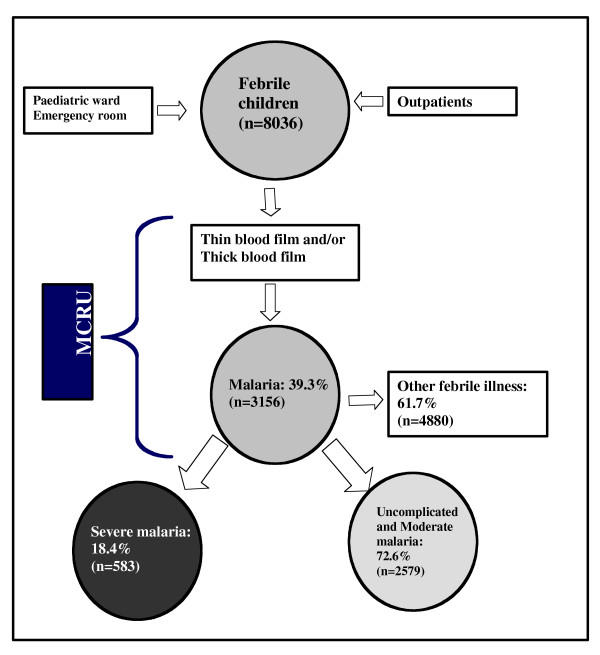
Schematic process of the screening of febrile children in the Centre Hospitalier de Libreville, Gabon.

Febrile children were referred the study team and seen on admission by a Malaria Clinical Research Unit (MCRU) clinician, summary data were recorded on a *pro-forma *sheet. A blood sample (2 ml) was drawn (anti-coagulated with EDTA) for quantitative examination of a blood film for malarial parasites using the Lambaréné method [11], measurement of haemoglobin concentration, white cell count, platelet count (STKS, Coulter Corporation) and blood lactate and glucose concentrations. Within 15 minutes of blood sampling, blood lactate and glucose concentrations were measured using Accusport™ hand held analyser (Bohringer, Manheim, Inc., Germany) and One-touch Analyzer (LifeScan, Inc., USA) [11]. Blood films were defined as negative if there were no asexual forms of *P. falciparum *in 100 high power fields of a thick film. The schematic process of the inclusion is shown in Figure 1.

Febrile children (or those with history of fever in the last 48 hours) were considered for inclusion in the study if they were: aged 0 to 10 years of age (inclusive), had malaria (> 2 asexual forms of *P. falciparum *seen on blood film) and had one or more of the following features of severity: [9,12,13]: Blantyre coma score (BCS) ≤ 2 defining cerebral malaria, repeated observed seizures (3 or more observed in 24 hours), lactate concentration in whole blood or capillary blood ≥ 5 mmol/L, glucose concentration in whole blood or capillary blood ≤ 2.2 mmol/L, severe anaemia (haemoglobin concentration of < 5 g/dL) and/or haematocrit concentration < 15%). Children were excluded from the study if informed consent was not obtained from a relative or if an alternative diagnosis was made clinically or by investigation (such as cerebrospinal fluid examination, chest radiography or blood culture).

Respiratory distress was defined as the presence of one or more of these features [14]: abnormalities in respiratory rate (according to the age), rhythm (Kussmaul's or Cheyne-Stokes's breathing) and signs of distress such as nasal flaring, intercostal or subcostal recession.

### Management

All children enrolled were hospitalised and treated with parenteral quinine (12.5 mg salt/kg/day, intravenous; Quinimax*, Sanofi-Synthelabo, France) without a loading dose, followed by oral quinine when tolerated. Pyrexial children received paracetamol suppositories (60 mg/kg/day, rectal; Efferalgan™, Bristol- UPSA, France). Seizures were controlled with diazepam (0.3 mg/kg, iv or 0.5 mg/kg rectal; Valium™, Roche, France). Severe anaemia was corrected by transfusion of packed red cells (15 ml/kg over 4 hours) screened for blood borne infections. Hypoglycaemia was treated with a slow intravenous injection of hypertonic glucose 40 %(Braun, Germany) at a dose of 1 ml/kg. Nasal oxygen at 6 1/minute was given to children with respiratory distress.

### Follow up

An MCRU clinician performed a full physical examination daily until discharge for each child. Laboratory assessments including parasitaemia, blood glucose and lactate were performed during hospitalization as necessary. The outcome (survived, death) was recorded.

### Statistical analysis

Statistical analysis was carried out with Epi info 6.04 (ENSP-Epiconcept- InVS, Corp.) and Stata Statistical Software (version 7.0, Stata Corporation, College Station, Texas, USA). Normality of data distribution was checked using either Shapiro-Wilks or Kolmogrov -Smirnov test. Normally distributed data were analysed by two-tailed Student's T test and non-normally distributed data with the Mann-Whitney U statistic. Proportions were compared with χ^2 ^tests with Yates' correction or Fisher's exact test. ANOVA test were used for multiple comparisons of variances, with Tukey's *post hoc *test. Assessments of prognostic factors were conducted with logistic regression model. A *p *value < 0.05 was considered as significant. Specific prevalence for each subgroup has been defined as the ratio of the number of cases observed in this sub-group over the population of this same sub-group. Hyperlactataemia is usually defined as a blood lactate concentration of ≥ 5 mmol/L. As the Accusport has been found to have poor agreement with the gold standard YSI 2300 [11] or YSI 1500 sport [15], a definition of hyperlactataemia as blood lactate concentration ≥ 10 mmol/L was used to increase the specificity in the analysis.

## Results

### Demographic and clinical data

During the study period (1st August 2000-31st July 2002), 8,036 febrile children were screened for malaria at the hospital. The data for 7,980 of these children were analysed: 4,368 male (54.5%) and 3,612 female (45.5%). Seventy-five percent these were less than 5 years of age. 3,156 (39.3%) of the 8,036 febrile children screened in the MCRU had a positive blood film for *P. falciparum*. A lower prevalence of malaria was seen in children aged< 6 months (3.7%, n = 118, p < 0.001). Specific prevalence of malaria rises after the 6 first months of life until it reaches a maximum at 47 months (47.5%), after which it declines again. Severe anaemia was most frequent in children less than 24 months old with 68 % of the cases of severe malarial anaemia occurring before this age. In contrast, the highest specific prevalence of cerebral malaria was found in children aged > 12 months. Sixty five percent of cases of cerebral malaria occurred in children aged between 12–48 months.

### Characteristics of severe malaria

**Figure 2:**
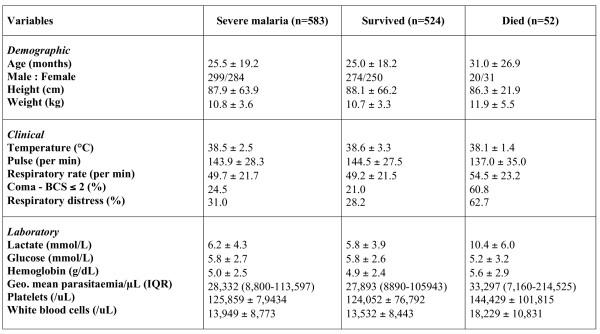
Admission characteristics of the study population (All results are mean ± SD except those specified.

The admission clinical, laboratory and parasitological characteristics of the 583 children with severe malaria are shown in the Figure 2. Two hundred and ninety nine of the severe malaria cases were male (51.3%) and 536 (92%) of the children with severe malaria were less than 5 years old. A history of vomiting, seizures and anti-malarial treatment before admission were reported in 322 (55.2%), 267 (45.8%) and 315 (54%) of the children with severe malaria respectively. Despite significant fluctuations in rainfall, the number of malaria cases per month was sustained during the study period, confirming the perennial transmission of malaria in this region.

Severe anaemia was the commonest feature of severe malaria present in 395 (67.8%,) of the children. Neurological presentations (either coma or repeated convulsions) were present in 228 (39.2%) of the children. Respiratory distress occurred in 181 (31%) of the children ; hyperlactataemia in 73 (15.7%) and hypoglycaemia in 33 (6.2%) of the children with severe malaria. Hyperparasitaemia (> = 20% of circulating infected red blood cells) was relatively rare, occurring in only 24 (4.1%) of the children with severe malaria.

Renal failure, acute pulmonary oedema and spontaneous bleeding are uncommon complications of childhood malaria [5,9,10] and were not seen in this study. Circulatory collapse was not found alone in this study and was not considered in the statistical analysis. One hundred and forty six (25%) children with severe malaria were afebrile on admission to hospital.

### Case rate fatality

**Figure 3:**
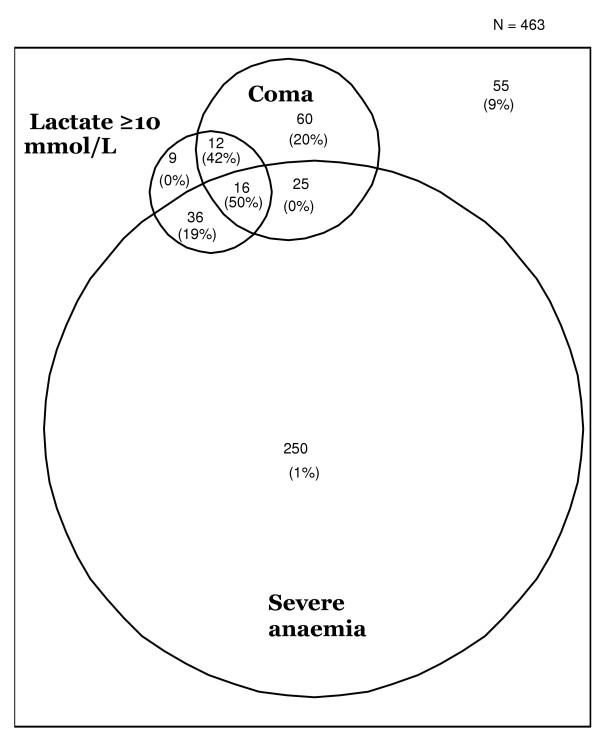
Venn diagram of features of 463 cases with a complete dataset of clinical measures. Case fatality rates shown in brackets.

Figure 3 shows a Venn diagram summarizing clinical and laboratory features of severe malaria and case fatality rates.

Of 583 patients, 52 died (40% male), giving an overall case fatality rate of 8.9%. Seven children were lost to follow-up. Forty seven deaths (90%) occurred within the first 24 hours after admission.

Neurological sequelae were present in 27 (5 %) of the 531 survivors. The case fatality rate was significantly higher in females than males (10.9% vs.6.9%, p = 0.006). Children who died were older than those who survived (mean (SD) = 35.4 (41.9) vs. 25.0 (18.2) months, p = 0.0009).

Of the 52 deaths in the course of the study, 32 (61.5%) presented with cerebral malaria, 32 (61.5%) presented with respiratory distress, and 20 (50% of the 40 with measurements) had hyperlactataemia.

Thirty (60%) of the children who died presented with convulsions, and 13 (25%) with hypoglycaemia. Twenty four (46.2%) of those who died had severe anaemia. Mortalities in each sub-group demonstrated that hyperparasitaemia (1 death in 24, 4%) and severe anaemia (24 deaths in 395, i.e. 6%) had a better prognosis than cerebral malaria (32 deaths in 142, i.e. 22.5%) and hypoglycaemia (15 deaths in 60, i.e. 25%). Thirty-two (17.7%) of the 181 patients with RD died, as did 30 (11%) of the 264 patients with convulsions and 20 (27.4 %) of the 73 patients with hyperlactataemia.

**Figure 4:**
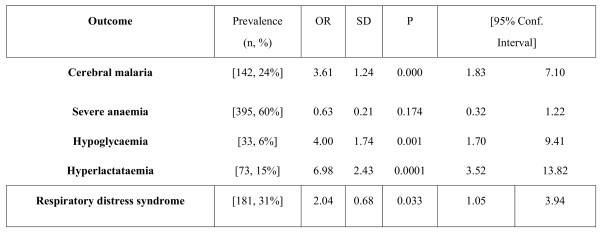
Prognostic indicators at the time of admission. OR – odds ratio, SD – standard deviation.

A multiple logistic regression model identified coma (OR = 3.6, 95% CI = 1.8–7.1, p < 0.001), hyperlactataemia (OR = 6.98, 95% CI = 3.5–13.8, p = 0.0001), respiratory distress (OR = 2.0, 95% CI = 1.0–3.9, p = 0.033) and hypoglycaemia (OR = 4.0, 95% CI = 1.7–9.4, p = 0.001) as independent predictors of a fatal outcome. Severe anaemia, hyperparasitaemia and thrombocytopaenia were not shown to be predictors of death (Figure 4).

## Discussion

Malaria remains a serious health problem in sub-Sahara Africa. It was the most common reason for neurological emergencies during 2001 at the CHL [7]. This study was designed to describe the epidemiology, clinical and laboratory presentations of severe falciparum malaria in childhood presenting to CHL, in order to improve the diagnosis, classification and appropriate management of malaria. It is not possible to exclude absolutely all children with alternative diagnoses on clinical examination and simple investigation alone, which is a problem shared by all other similar studies on severe malaria [3-5,9,10,14,16-18]. The small numbers with alternative diagnoses should not affect the conclusions of this or other studies. Most cases (92%) of severe malaria were in children less than 5 years old. Similar observations have been made in another group of children hospitalized for malaria in Gabon [19]. Elsewhere, severe malaria tends to occur in older children [20]. Differences in the age of presentation of severe malaria may be the result of lower background immunity or other undefined variables [21]. The study confirms the stable and perennial transmission of malaria in Gabon, which contrasts with reports from other countries in West Africa where malaria transmission is predominantly at the end of the long rainy season [22,23].

Fever is a characteristic feature of *P. falciparum *infection, but a sizeable proportion of these children (25%) with severe malaria were afebrile on admission as observed elsewhere [23]. Self-medication with antipyretic or antimalarial agents was common (about 50% of the children) and may contribute to this finding. There are obvious implications for the diagnosis of malaria, which may be underestimated using clinical criteria alone.

Severe anaemia was the most frequent feature of severity in this study, but was associated with decreased mortality. A similar observation in a recent Ghanaian study showed a better outcome in children with severe anaemia [17]. These findings confirm that severe malaria anaemia has a lower case fatality rate than other complications of severe malaria, consistent with several other studies where severe anaemia was not an independent predictor of in-hospital mortality [9,10,18]. The case fatality rate of severe anaemia without other markers of severe malaria is 1 to 2%, where blood transfusion is available [3,9,10,14,18] raising questions about the value of severe anaemia as a defining feature of the syndrome of severe malaria.

Despite the increasing toll of HIV infection, and the continuing burden of diarrhoeal disease, malnutrition and respiratory tract infections, malaria remains a major cause of childhood death in endemic regions [1]. The overall case fatality rate of severe malaria in the study was 8.9% (52 deaths/583 cases), which is in keeping with studies from other geographic areas, where case fatality rates range between 8 and 40% [4,5,9,14,16,20,22,24,25]. Most of these deaths (90% in this study) occurred in the first twenty-four hours of hospital admission, a finding also in keeping with other studies [5].

The independent prognostic indicators in this study were cerebral malaria, respiratory distress, hypoglycaemia and hyperlactataemia. These observations are entirely consistent with a large number of studies where the independent predictors of a fatal outcome in malaria are impaired consciousness and metabolic dysfunction (as measured by hyperlactataemia, hypoglycaemia, acidosis or respiratory distress) [3,5,9,10,14,16-18]. The metabolic complications of malaria are complex and a number of interrelated measures have been used in different studies. Severe malaria is associated with a metabolic acidosis [16] and hyperlactataemia [5]. Respiratory distress has been associated with acidosis and hyperlactataemia in some studies [26]. These features of metabolic malaria probably all result from increased anaerobic metabolism due to tissue hypoxia [27].

Estimates of the prevalence of hypoglycaemia have been reported in Africa, ranging from 8% to 34% [28,29]. In severe childhood malaria hypoglycaemia results from impaired gluconeogenesis and increased tissue demand for glucose [27,28] and quinine induced hyperinsulinaemia. Blood glucose concentrations should be monitored in all children hospitalised for malaria especially those who receive quinine.

The definition of hyperlactataemia used in this study was a blood lactate concentration higher than the conventional cut-off (≥ 5 mmol/L). This was necessary because of the limitations of the analyser used, but probably means that the frequency of true hyperlactataemia was underestimated. The Accusport™ analyser used has been shown to have poor agreement with "gold standard" machines [11,15,30].

Hyperlactataemia is a frequent and serious complication of severe malaria in childhood [5,9,10], which may be due microcirculatory sequestration of parasitized erythrocytes resulting in increased production of lactate by anaerobic glycolysis [31]. A recent study showed a correlation between hyperlactataemia and high plasma glutamine levels in severe malaria. This correlation may reflect impaired gluconeogenesis [31]. Lactate disposal is proportional to blood lactate concentration and can be increased by dichloroacetate [27,32]. Lactic acidosis, as confirmed in this study, is an established strong predictor of a fatal outcome in falciparum malaria in African children [5,9,10] and may prove a target for further interventional studies to improve survival. Respiratory distress was present in 31% of these children. This is higher than the frequency reported in other studies of severe malaria: 4.9% in Burkina [20], 6.4% in Togo [22] and 13.7% in Kenya [14].

These differences may partly be explained by low inter-observer agreement for this variable, geographical variations in disease pattern as well as differing definitions of severe malaria. Results from many studies consistently show that respiratory distress is a life-threatening syndrome in childhood malaria [14,33]. Respiratory distress was significantly associated with both hyperlactataemia and cerebral malaria in this study. The Blantyre coma score has long been established in children as a good indicator of cerebral dysfunction in malaria [3] and has enabled better standardization of studies on cerebral malaria in African children. The case-fatality rate associated with cerebral malaria (22%) is similar to that in Gambian children (27%) [4] but is higher than that observed for Kenyan (17%) [14] and Malawian children (15%) [3]. It has been postulated that with the higher the level of malaria transmission, immunity is acquired earlier, perhaps altering the presentation of severe malaria from predominantly a cerebral syndrome to that of severe anaemia [34,35].

The clinical and laboratory presentations of severe malaria are described in a hospitalized population of children in Gabon. The severe cases are likely to be only the "tip of the iceberg", many children living far from health care units may die whilst travelling to the nearest hospital. Most deaths from malaria occurred in the first 24 hours of admission, which highlights the need for early recognition of the most severely ill children. Early diagnosis and classification of severe malaria would allow appropriate management, including basic adjunctive therapy such as to prevent hypoglycaemia, and better use of scarce healthcare resources. Together these improvements could contribute to a reduction in the intractably high mortality due to the disease.

## Authors' contributions

AD is a MCRU clinician. He participated to the study and wrote the article. PN conducted the study for his MD thesis. RT and MB are paediatricians who participated in the study. TP is a UK collaborator who participated in the study and helped write the article. MM was involved with the intensive care of the children. UMR participated in the study as a MCRU clinician. JJ helped to write the article. EK did all the statistical analysis. ENM, PK, SK and MK coordinated the realization of the study and edited the final version approved by all authors.
